# Retrospective Analysis of the Correlation between Uric Acid and Thyroid Hormone in People with Normal Thyroid Function

**DOI:** 10.1155/2019/5904264

**Published:** 2019-07-07

**Authors:** Guanqun Chao, Yue Zhu, Lizheng Fang

**Affiliations:** Department of General Practice, Sir Run Run Shaw Hospital, Zhejiang University, China

## Abstract

**Objective:**

This study adopts the method of retrospective analysis to collect general information and laboratory results of physical examination population, hoping to clarify the correlation between uric acid and thyroid hormone.

**Methods:**

The subjects of the study were healthy subjects who underwent physical examination at the Sir Run Run Shaw Hospital affiliated to the Medical College of Zhejiang University from January 2016 to December 2018. Demographic information and medical history of all subjects were recorded through an electronic health system. Serum uric acid (SUA) was grouped by quartiles. Statistical analyses were performed with R version 3.5.1.

**Results:**

A total of 48,526 subjects were included in the analysis. Gender ratio, age, BMI, waist circumference, systolic blood pressure, diastolic blood pressure, FBG, HbA1c, TG, HDL-C, ALT, AST, FT3, FT4, and TSH were significantly different among the uric acid groups. The regression coefficients of SUA in the TSH, FT3, and FT4 regression models were *B* = 1.000 (95% CI 1.000-1.000, *p* = 0.009), *B* = 0.999 (95% CI 0.999-0.999, *p* < 0.001), and *B* = 1.001 (95% CI 1.001-1.001,  *p* < 0.001), respectively. There was a significant dose-dependent relationship between FT4, FT3, and SUA gradient.

**Conclusions:**

Under normal thyroid function, there were significant differences in TSH, FT3, and FT4 between groups with different uric acid levels. Uric acid levels were linearly correlated with FT3 and FT4, but not with TSH.

## 1. Introduction

Currently, some studies have pointed out that the correlation between thyroid hormone and uric acid level, but this correlation is still controversial. A recent study suggests that thyroid hormone may regulate uric acid levels in patients with subclinical hypothyroidism by regulating insulin resistance [1]. In a study of hyperuricemia, hypothyroidism was found to be a risk factor for men more than women, leading the researchers to consider the effects of sex hormones [2]. The differences in research directions lead to the discovery of more new correlations, and these studies have also provided us with effective clues and solutions to solve practical problems in clinical practice.

Uric acid is mainly produced by the liver, and it is a water-soluble antioxidant [3]. Uric acid has been shown to directly inhibit the damage which is caused by free radicals and also to protect cell membranes and DNA [4]. Uric acid is a heterocyclic compound composed of hydrogen, carbon, oxygen, and nitrogen [5]. Uric acid levels were found to be associated with benign positional vertigo, but were not an independent risk factor [6]. A study has also suggested that plasma uric acid levels are associated with platelet reactivity in the elderly [7]. The uric acid level increase is believed to be an intermediary factor in adipose tissue that regulates endocrine disorders that promote inflammation and may be an important factor leading to dyslipidemia and atherosclerosis [8]. At present, it can be seen that there are many studies on uric acid, and uric acid is also found to be related to cardiovascular disease, kidney disease, etc., but there are a few studies on the correlation between uric acid and thyroid function, and also, there are some controversies.

A recent study suggests that thyroid hormones and thyroid stimulating hormone (TSH) are associated with the function of each organ system but also affect the body's growth and development, and it was found that the concentration of the thyroid hormones is owing to different age and sex [9]. Another study has found that thyroid hormones play a central regulatory role in the cardiovascular system and are considered a target for the treatment of heart failure [10]. At present, it is believed that thyroid hormone changes have a certain heritability, but most of the genetic possibilities cannot be explained; also, an analysis has found FT3-related genome-wide variations and new TSH-related loci [11]. It can be seen that studies on thyroid function have gone deep into epigenetics, but the correlation between thyroid function and uric acid is still controversial and cannot be better explained. Therefore, this study adopts the method of retrospective analysis to collect general information and laboratory results of physical examination population, hoping to clarify the correlation between uric acid and thyroid hormone.

## 2. Methods

### 2.1. The Research Object

The subjects of the study were healthy subjects who underwent physical examinations at the Sir Run Run Shaw Hospital affiliated to the Medical College of Zhejiang University from January 2016 to December 2018. They were aged between 18 and 80.

### 2.2. Excluded Objects

The following are the exclusion criteria:

1. Patients with severe heart, liver, and kidney dysfunction
2. Individuals with a history of thyroid disease, including those previously diagnosed with apparent hypothyroidism or hyperthyroidism, thyroid cancer or thyroid nodules, thyroid hormone/antithyroid drug intake history, or previous thyroid surgery or radioactive iodine intake
3. Patients with hyperuricemia or gout who continue to receive medication

The study was approved by the ethics committee of Sir Run Run Shaw Hospital affiliated to Zhejiang University.

### 2.3. Indicator Collection

Demographic information and medical history of all subjects were recorded through an electronic health system. Laboratory indicators include fasting blood glucose (FBG), total cholesterol (TC), total triglycerides (TG), low-density lipoprotein cholesterol (LDL-C), high-density lipoprotein cholesterol (HDL-C), HbA1c, alanine aminotransferase (ALT), aspartate aminotransferase (AST), free triiodothyronine (FT3), free thyroxine (FT4), thyroid-stimulating hormone (TSH), and serum uric acid (SUA). Normal TSH levels were 0.35-4.94 mIU/L, normal FT3 levels were 1.71-3.71 pg/mL, and normal FT4 levels were 0.7-1.48 ng/dL. The thyroid function of each subject was classified as subclinical hyperthyroidism (TSH < 0.40 mIU/L, normal FT4), subclinical hypothyroidism (TSH > 4.00 mIU/L, normal FT4), or euthyroidism (TSH of 0.40-4.00 mIU/L) based on previously established criteria. Serum uric acid was grouped by quartiles, including Q1 (UA < 283 mol/L), Q2 (283-345 mol/L), Q3 (345-410 mol/L), and Q4 (>410 mol/L).

### 2.4. Statistical Analysis

Descriptive analyses are expressed as the mean ± standard deviation (SD) for continuous variables, while qualitative variables were expressed as percentages (count). The analysis of variance (ANOVA) was used to compare serum TSH, FT3, and FT4 levels among SUA groups. All statistical tests were carried out in a two-sided manner, and *p* < 0.05 was considered statistically significant. Statistical analyses were performed with R version 3.5.1.

## 3. Results

### 3.1. General Data Analysis

**Figure 1:**
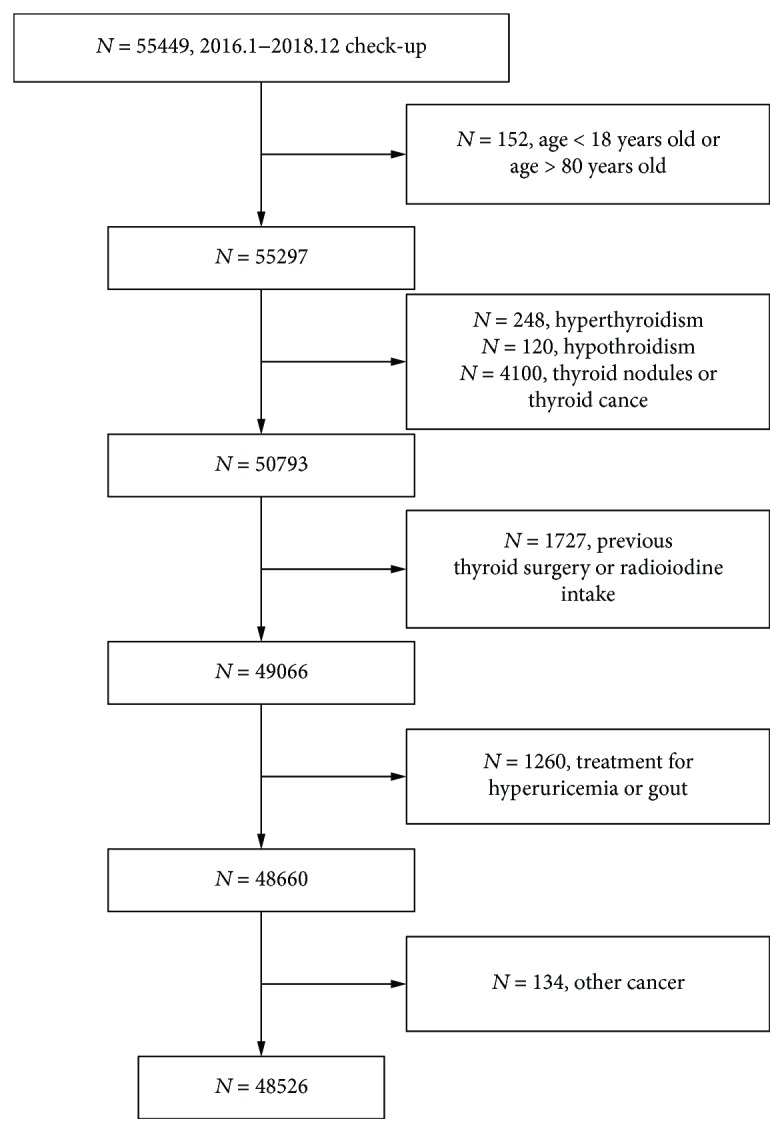
Flow chart of the study population.

Finally, according to the inclusion principle and exclusion criteria, a total of 48,526 subjects were included in the analysis (Figure 1). Gender ratio, age, BMI, waist circumference, systolic blood pressure, diastolic blood pressure, FBG, HbA1c, TG, HDL-C, ALT, AST, FT3, FT4, BUN, creatinine, and TSH were significantly different among the uric acid groups (*p* < 0.05) (see Table 1).

### 3.2. Linear Regression Analysis

In order to better determine the correlation between the level of metabolic indicators and TSH, FT3, and FT4, we conducted a linear regression analysis. Gender, age, BMI, waist circumference, blood pressure, FBG, blood lipid, liver function, SUA, and other related metabolic indexes were included in the multiple linear regression analysis. The results showed that after adjusting the gender, age, BMI, SBP, DBP, FBG, TC, TG, LDL-C, HDL-C, and SUA were significantly correlated with TSH, FT3, and FT4. The regression coefficients of SUA in the TSH, FT3, and FT4 regression models were *B* = 1.000 (95% CI 1.000-1.000, *p* = 0.009), *B* = 0.999 (95% CI 0.999-0.999, *p* < 0.001), and *B* = 1.001 (95% CI 1.001-1.001, *p* < 0.001), respectively (Table 2), suggesting that SUA could affect the levels of TSH, FT3, and FT4.

### 3.3. Multivariate Linear Regression Analysis

In order to further explore the effects of SUA with different levels of gradient on TSH, FT3, and FT4, we took the SUA interval Q1-Q4 as the dependent variable and analyzed it together with other metabolic indicators such as gender and age. Multivariate linear regression analysis shows that there was a significant dose-dependent relationship between FT3 and SUA gradients (multivariate linear analysis showed that FT3 and FT4 were statistically significantly associated with the grade of SUA in a dose-dependent manner) (*B* 0.965, 95% CI 0.939-0.993 in Q2; *B* 0.887, 95% CI 0.855-0.919 in Q3). There was a significant dose-dependent relationship between FT4 and SUA gradients (*B* 1.101, 95% CI 1.033-1.174 in Q2; *B* 1.230, 95% CI 1.133-1.335 Q1, *p* for trend < 0.01) (Table 3).

## 4. Discussion

In this retrospective analysis, we included data from 48,526 subjects who underwent physical examination. We found that there were significant differences in the levels of FT3, FT4, and TSH between different uric acid levels. In the linear correlation analysis, it was confirmed that there was a linear correlation between FT3, FT4, and TSH and uric acid level. In further multivariate linear regression analysis, we found that FT3 and FT4 were correlated with uric acid, but TSH was not.

For several years, researchers have conducted cross-sectional studies on thyroid function and uric acid levels, but the results were varied and controversial, which is why we conducted this retrospective analysis. Some researchers have proposed that hyperuricemia and gout can increase the prevalence of hyperthyroidism, and a further study has found that the uric acid level in hyperthyroidism patients is significantly higher, but lower than that in hypothyroidism patients [12]. As early as 1955, researchers proposed that hypothyroidism was associated with hyperuricemia [13], but subsequent studies found a contradictory relationship between thyroid dysfunction and uric acid. Another cross-sectional study found a close correlation between uric acid levels and thyroid function, but no correlation between hyperuricemia and thyroid function [14]. It also suggested that there was a linear correlation between FT4 and uric acid levels in the population without thyroid dysfunction, and the incidence of hyperuricemia increased with the increase of FT4 [15]; this result is consistent with our findings. Saini et al. found that TSH was positively correlated with the uric acid level in hypothyroidism patients, while FT4 was negatively correlated with the uric acid level [16]. Similarly, in the study on hypothyroidism patients, TSH was found to be positively correlated with uric acid, while FT4 was not correlated with uric acid [17]. Thus, it can be seen that there are consistent and contradictory results in multiple related studies. According to the previous literature and research results, we analyzed the reasons for these contradictory results as follows: (1) the population of the study was classified differently, some were patients with thyroid dysfunction, and some were normal population; (2) there are differences in reagents tested in the laboratory, leading to differences in results; (3) different grouping methods and statistical methods lead to differences in analysis results. Therefore, our study excluded patients with thyroid disorders and those taking drugs affecting thyroid hormone and uric acid levels and selected healthy people for physical examination as the main research objects.

Uric acid levels are affected by many factors. A recent study suggests that triglycerides are an independent risk factor for hyperuricemia, with higher triglycerides associated with higher uric acid levels [18]. In a study of men with type 2 diabetes, low uric acid levels were associated with an increased incidence of major nerve fiber dysfunction [19]. In a study of healthy people, lower than normal levels of uric acid in the blood can increase the risk of hypertension [20]. Hyperuricemia is also considered a risk factor for intracranial artery stenosis in elderly patients with cerebral infarction [21]. Increased uric acid is thought to increase the incidence of kidney disease, and decreased uric acid helps reduce the development of kidney disease [22]. A recent study suggests that uric acid may be an important biomarker of cell necrosis rather than an antioxidant [23]. Hyperuricemia and gout are significantly correlated with genetic heritability and support the correlation with the SLC28A2 gene [24]. Hyperuricemia has been found to be associated with age and gender, suggesting that among obese and overweight people, young people are more likely to develop hyperuricemia than older people, while women are more likely to develop hyperuricemia as they gain weight [25]. As we know, uric acid is excreted primarily by the urine through the kidneys. Xanthine oxidase and xanthine dehydrogenase mainly act on the metabolism and production of uric acid. Another study found that increased uric acid was associated with chronic kidney disease, as well as pulmonary vascular obstruction, and inhibition of xanthine oxidase is the key mechanism [26]. In conclusion, the increase in uric acid has been linked to a variety of diseases; the uric acid level is affected by many factors, including genes, age, and gender. However, according to various studies, the uric acid level is not the higher the better nor the lower the better. Although our study confirmed that there was a linear correlation between FT3 and FT4 and uric acid levels, it was not necessary to strictly regulate uric acid levels for thyroid hormone levels nor to regulate thyroid hormone levels for uric acid levels.

Our study still has some limitations. On the one hand, our study excluded patients with thyroid disease and patients taking drugs that affect thyroid hormone and uric acid levels. This is the advantage of our study, but we cannot exclude other influencing factors such as diet and region. (1) We did not further analyze the correlation between thyroxine and time lapse, so as to cause the effect on uric acid. (2) Because the data used for the analysis were obtained from the results of the health examination and they were not detected for urinary excretion of uric acid, no further analysis is possible. In conclusion, our study can show that under normal thyroid function, there is a close relationship between different uric acid levels and TSH, FT3, and FT4 levels. Further analysis confirmed that the uric acid level was linearly correlated with FT3 and FT4, but not with TSH. In conclusion, the metabolism and production of uric acid mainly pass through the metabolic action of xanthine oxidase and xanthine dehydrogenase. Low oxygen, inflammation, etc., can accelerate enzyme metabolism and cause changes in the uric acid level, thereby causing the production of related cytokines. Therefore, we speculate that thyroid hormones can also change the level of cytokines produced by oxidative stress and inflammation, and the change of thyroid hormones can also cause the production of related cytokines and the change of enzyme level and finally affect the uric acid level. We hypothesized that FT3 and FT4 effects on uric acid may be through the transformation of purine nucleotides and uric acid excretion; therefore, further prospective studies are needed to confirm these findings.

## 5. Conclusion

Uric acid levels are correlated with FT3, FT4, and TSH and are linearly correlated with FT3 and FT4. The reason may be that thyroid hormones affect uric acid levels by affecting the conversion of purine nucleotides and excretion of uric acid.

## Figures and Tables

**Table 1:** Characteristics of subjects stratified according to the quartiles of uric acid (mean ± SD).

	Uric acid				
	<283 (*n* = 4705)	283~ (*n* = 35723)	345~ (*N* = 7970)	410~ (*n* = 128)	*p* value^a^
Male gender (%)	361 (7.7%)	20254 (56.7%)	7689 (96.5%)	124 (96.9%)	<0.001
Age (years)	44.8 ± 9.70	46.9 ± 10.5	46.1 ± 10.3	44.7 ± 11.1	<0.001
BMI (kg/m^2^)	22.1 ± 2.88	23.9 ± 3.19	25.7 ± 3.16	26.9 ± 3.17	<0.001
Waist circumference (cm)	74.8 ± 8.11	83.3 ± 10.0	91.2 ± 8.18	94.8 ± 8.22	<0.001
Blood pressure (mmHg)					
Systolic blood pressure	115 ± 16.3	122 ± 16.4	128 ± 14.6	130 ± 13.5	<0.001
Diastolic blood pressure	67.9 ± 10.5	72.9 ± 11.1	78.2 ± 10.9	80.4 ± 9.88	<0.001
FBG (mmol/L)	5.19 ± 1.29	5.34 ± 1.22	5.44 ± 0.989	5.65 ± 0.912	<0.001
Hba1c (%)	5.33 ± 0.821	5.43 ± 0.759	5.44 ± 0.651	5.48 ± 0.709	<0.001
Triglycerides (mmol/L)	1.08 ± 0.718	1.65 ± 1.40	2.55 ± 1.98	3.35 ± 2.44	<0.001
Total cholesterol (mmol/L)	4.60 ± 0.896	4.80 ± 0.943	5.03 ± 0.986	5.16 ± 0.974	<0.001
HDL-cholesterol (mmol/L)	1.41 ± 0.308	1.22 ± 0.307	1.06 ± 0.242	1.01 ± 0.235	<0.001
LDL-cholesterol (mmol/L)	2.52 ± 0.710	2.73 ± 0.757	2.82 ± 0.797	2.75 ± 0.833	<0.001
Alanine aminotransferase (IU/L)	16.4 ± 11.5	24.5 ± 24.2	36.2 ± 27.1	48.7 ± 49.7	<0.001
Aspartate aminotransferase (IU/L)	18.5 ± 7.09	21.3 ± 12.2	25.4 ± 14.2	30.1 ± 18.8	<0.001
BUN (mmol/L)	4.43 ± 1.14	4.76 ± 1.14	4.95 ± 1.14	5.06 ± 1.19	<0.001
Creatinine (*μ*mol/L)	58.61 ± 10.48	66.97 ± 12.84	75.51 ± 12.75	81.12 ± 16.63	<0.001
TSH (mIU/L)	1.98 ± 1.79	1.84 ± 1.51	1.77 ± 1.08	1.75 ± 0.984	<0.001
FT3 (pg/mL)	2.54 ± 0.795	2.62 ± 0.883	2.64 ± 0.901	2.59 ± 0.860	<0.001
FT4 (ng/dL)	2.15 ± 1.97	2.18 ± 1.99	2.24 ± 2.00	2.34 ± 2.06	0.049

^a^Analysis of variance (ANOVA) was used to compare serum TSH, FT3, and FT4 levels among UA grades.

**Table 2:** Results of multiple linear regression of TSH, FT3, and FT4.

	TSH		FT3		FT4	
	*B* (95% CI)	*p* value	*B* (95% CI)	*p* value	*B* (95% CI)	*p* value
Male gender	0.653 (0.629-0.678)	<0.001	1.181 (1.155-1.207)	<0.001	0.938 (0.892-0.987)	0.013
Age	1.002 (1.000-1.003)	0.009	0.999 (0.998-1.000)	0.077	0.993 (0.992-0.995)	<0.001
BMI	1.009 (1.003-1.015)	0.003	0.993 (0.990-0.997)	<0.001	1.002 (0.994-1.010)	0.565
WC	1.000 (0.998-1.002)	0.913	1.005 (1.004-1.007)	<0.001	0.995 (0.992-0.999)	0.004
SBP	1.001 (0.999-1.002)	0.404	1.001 (1.001-1.002)	<0.001	1.004 (1.002-1.005)	<0.001
DBP	1.003 (1.001-1.005)	0.002	1.000 (0.999-1.001)	0.064	1.001 (0.998-1.003)	0.575
FBG	0.972 (0.961-0.983)	<0.001	1.010 (1.003-1.017)	0.004	0.984 (0.968-0.999)	0.043
TG	1.007 (0.990-1.023)	0.424	0.918 (0.909-0.927)	<0.001	1.239 (1.213-1.267)	<0.001
TC	1.124 (1.080-1.171)	<0.001	1.345 (1.317-1.382)	<0.001	0.453 (0.429-0.478)	<0.001
HDL-C	1.002 (0.940-1.068)	0.945	0.757 (0.728-0.786)	<0.001	2.319 (2.128-2.527)	<0.001
LDL-C	0.936 (0.895-0.979)	<0.001	0.749 (0.729-0.770)	<0.001	2.256 (2.123-2.398)	<0.001
SUA	1.000 (1.000-1.000)	0.009	0.999 (0.999-0.999)	<0.001	1.001 (1.001-1.001)	<0.001

SBP: systolic blood pressure; DBP: diastolic blood pressure; WC: waist circumference; FBG: fast blood glucose; SUA: serum uric acid; TC: total cholesterol; TG: triglycerides; LDL-C: low-density lipoprotein cholesterol; HDL-C: high-density lipoprotein cholesterol; ALT: alanine aminotransferase; AST: aspartate aminotransferase; TSH: thyroid-stimulating hormone; FT3: free triiodothyronine; FT4: free thyroxine.

**Table 3:** Result of regression coefficients of TSH, FT3, and FT4 for the grade of uric acid.

	TSH		FT3		FT4	
	*B* (95% CI)	*p* value	*B* (95% CI)	*p* value	*B* (95% CI)	*p* value
UA quantile						
Q1	1.000	/	1.000	/	1.000	/
Q2	1.010 (0.963-1.059)	0.675	0.965 (0.939-0.993)	0.014	1.101 (1.033-1.174)	0.003
Q3	1.044 (0.982-1.109)	0.171	0.887 (0.855-0.919)	<0.001	1.230 (1.133-1.335)	<0.001
Q4	0.985 (0.760-1.276)	0.909	0.833 (0.715-0.971)	0.020	1.383 (0.976-1.959)	0.068

TSH: thyroid-stimulating hormone; FT3: free triiodothyronine; FT4: free thyroxine. The multivariate model was adjusted for age, gender, body mass index, systolic blood pressure, diastolic blood pressure, waist circumference, triglyceride, total cholesterol, high-density lipoprotein cholesterol, and low-density lipoprotein cholesterol, respectively.

## Data Availability

The data used to support the findings of this study are available from the corresponding author upon request.
